# Leveraging electrocardiography signals for deep learning-driven cardiovascular disease classification model

**DOI:** 10.1016/j.heliyon.2024.e35621

**Published:** 2024-08-05

**Authors:** Hamed Alqahtani, Ghadah Aldehim, Nuha Alruwais, Mohammed Assiri, Amani A. Alneil, Abdullah Mohamed

**Affiliations:** a Department of Information Systems, College of Computer Science, Center of Artificial Intelligence, Unit of Cybersecurity, King Khalid University, Abha, Saudi Arabia; b Department of Information Systems, College of Computer and Information Sciences, Princess Nourah Bint Abdulrahman University, P.O. Box 84428, Riyadh, 11671, Saudi Arabia; c Department of Computer Science and Engineering, College of Applied Studies and Community Services, King Saud University, Saudi Arabia, P.O.Box 22459, Riyadh, 11495, Saudi Arabia; d Department of Computer Science, College of Sciences and Humanities- Aflaj, Prince Sattam bin Abdulaziz University, Aflaj, 16273, Saudi Arabia; e Research Centre, Future University in Egypt, New Cairo, 11845, Egypt

**Keywords:** Healthcare, ECG signals, Cardiovascular disease, Deep learning, Pattern recognition, Parameter tuning

## Abstract

Electrocardiography (ECG) is the most non-invasive diagnostic tool for cardiovascular diseases (CVDs). Automatic analysis of ECG signals assists in accurately and rapidly detecting life-threatening arrhythmias like atrioventricular blockage, atrial fibrillation, ventricular tachycardia, etc. The ECG recognition models need to utilize algorithms to detect various kinds of waveforms in the ECG and identify complicated relationships over time. However, the high variability of wave morphology among patients and noise are challenging issues. Physicians frequently utilize automated ECG abnormality recognition models to classify long-term ECG signals. Recently, deep learning (DL) models can be used to achieve enhanced ECG recognition accuracy in the healthcare decision making system. In this aspect, this study introduces an automated DL enabled ECG signal recognition (ADL-ECGSR) technique for CVD detection and classification. The ADL-ECGSR technique employs three most important subprocesses: pre-processed, feature extraction, parameter tuning, and classification. Besides, the ADL-ECGSR technique involves the design of a bidirectional long short-term memory (BiLSTM) based feature extractor, and the Adamax optimizer is utilized to optimize the trained method of the BiLSTM model. Finally, the dragonfly algorithm (DFA) with a stacked sparse autoencoder (SSAE) module is applied to recognize and classify EEG signals. An extensive range of simulations occur on benchmark PTB-XL datasets to validate the enhanced ECG recognition efficiency. The comparative analysis of the ADL-ECGSR methodology showed a remarkable performance of 91.24 % on the existing methods.

## 1 Introduction

Automatic analysis of ECG patterns helps early recognize life-threatening arrhythmias, namely ventricular tachycardia, atrial fibrillation, and atrioventricular block, and is highly useful for the clinician [1]. This system must use an algorithm to recognize distinct waveform varieties in ECG and identify complicated relationships among them. However, great diversity from wave morphology among the occurrence of noise and patients are the main problems [2]. The restriction of various approaches utilized to automated ECG classifiers cannot manage larger intraclass variation. They largely depend on supervised trained datasets and ineffectively perform while processing a considerable amount of ECG data. Furthermore, the reduction dimension method extracts features in the transform domain, greatly enhancing the overall operation's computational difficulty [3]. In addition, the classifier algorithm doesn't perform despite wide interpatient variation in ECG signals. Consequently, unreliable performances make classifier algorithms inconsistent in medical settings.

Cardiologists regularly employ computerized recognition of ECG abnormality by categorizing long-term ECG records [4]. The feature-extracting approach involves Hermite functions, waveshape functions, statistical features, and wavelet-based features. The current automatic ECG recognition technique frequently relies on pattern-matching infrastructure representing the ECG signals in order of stochastic patterns [5]. Complicated feature-extracting methods are needed, and high sampling rates are time-consuming. In order to provide real-time performance from the medical at reasonable costs, this technique utilizes a lower sampling rate and a simpler group of features [6].

The current ECG classification algorithm commonly includes signal pre-processing, namely manual feature extraction and wavelet transform. However, the number of computations increases the delay of the real-time classification method [7]. Recently, the DL approach, with the benefits of automated learning features, has been widely employed in healthcare, namely healthcare image segmentation and recognition, time series data analysis, and monitoring [8]. Now, the efficient method could establish an end-to-end deep neural network (DNN) for learning the features of ECG records by utilizing the wide-ranging digital features of ECG records that keep many signals during pre-processing phases. Since the implementation of DNN will increase the training data, this approach could utilize the wide-ranging digitization of ECG data [9]. The DL is a machine learning (ML) method that becomes conventional for pattern detection [10]. The DL approach has considerably improved the performance of recognition tools.

This study designs an automated DL enabled ECG signal recognition (ADL-ECGSR) technique for CVD detection and classification. The ADL-ECGSR technique employs four major pre-processed subprocesses: feature extraction, parameter tuning, and classification. In addition, the ADL-ECGSR method includes the design of a bidirectional long short-term memory (BiLSTM) based feature extractor, and the Adamax optimizer is utilized to improve the trained method of the BiLSTM method. Moreover, the dragonfly algorithm (DFA) with a stacked sparse autoencoder (SSAE) model is exploited to recognize and classify EEG signals. The performance analysis of the ADL-ECGSR method takes place against the benchmark PTB-XL dataset, and the outcomes are inspected under various measures.

The rest of the study is organized in this way: Section 2 provides a comprehensive analysis of the study, and section 3 discusses the presented method. Next, section 4 provides experimental validation, and section 5 concludes the study.

## 2 Literature review

Liu and Kim [11] presented a classification model of heart disease-based ECG by adapting an ML technique named long short-term memory (LSTM), which is an advanced method examining time series sequences in the DL approach. As appropriate data pre-processing, the model uses symbolic aggregate approximation (SAX) to enhance the performance. Wu et al. [12] presented an efficient and robust 12-layer deep 1D deep convolutional neural network (1D-DCNN) on categorizing the five micro-classes of heartbeat type from the MIT-BIH Arrhythmia dataset.

Hasan and Bhattacharjee [13] introduced an approach for classifying various heart diseases using 1D-DCNN in which adapted ECG signals are offered as input signals to networks. First, all the ECG signals are decomposed using Intrinsic Mode Functions (IMFs) and Empirical Mode Decomposition (EMD), which are integrated to form an adapted ECG signal. This processing signal is given to the convolution neural network (CNN), which categorizes the records based on CVD using a softmax regressor. Yıldırım et al. [14] designed a comprehensive end-to-end architecture rather than the handcrafted feature selection and extraction in conventional models. The major contribution is to develop a 1D-CNN. The presented methodology is 1) fast (real-time classification), 2) efficient, 3) easy to use (fused feature selection and extraction and classification in one phase), and 4) non-complex.

In Li et al. [15], the rhythm and morphology of heartbeats were combined into a 2D data vector for succeeding procedure by CNN that involves biased dropout and adaptive learning rate method. The outcome shows that the presented CNN method was effective in detecting irregular arrhythmias or heartbeats through automated feature extraction. Li et al. [16] constructed a CNN architecture spatial pyramid pooling (SPP) model that resolves the shortcomings created by the size of input information. The MIT-BIH arrhythmia dataset is applied as the testing and training information for classifying heartbeat signals into six classes. In comparison to the conventional technique, which might lose a considerable number of data and make it easier to be over-fitted, the strength of the presented technique is assured by extracting data features from distinct sizes.

## 3 The proposed model

**Fig. 1:**
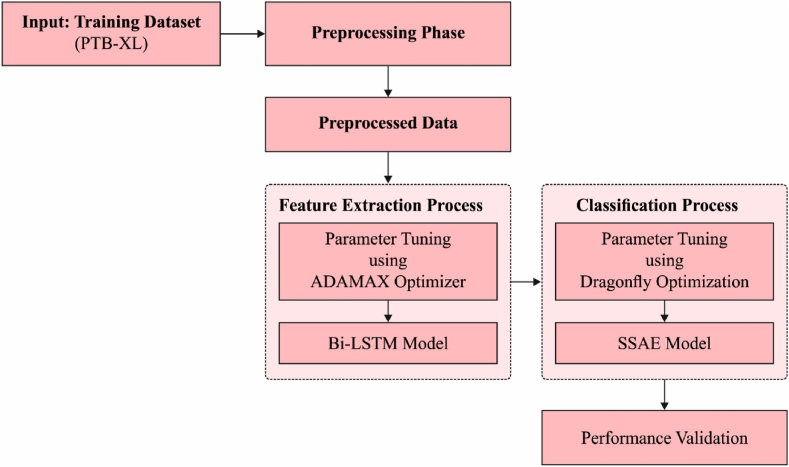
The working process of ADL-ECGSR method.

This study develops a novel ADL-ECGSR method for CVD detection and classification. The ADL-ECGSR technique incorporates different stages of operations namely pre-processing, BiLSTM based feature extraction, Adamax based hyperparameter optimization, DFA based parameter tuning, and SSAE based classification. The Adamax and DFA helps to considerably boost the ECG detection performance. Fig. 1 depicts the working process of presented ADL-ECGSR technique.

### 3.1 Data pre-processing

During pre-processing, a group of 3000 ECG records is regarded for experimental analysis. As 35 ECG record contains null value as class label, they can be thrown out in the dataset and overall, 2965 ECG record is utilized for experiment analysis. Also, a sampling rate of 100 has been selected amongst 2 sampling rates of 100 and 500 in the datasets, for the research.

### 3.2 Design of optimal BiLSTM model

In the feature extraction procedure, the BiLSTM method was exploited to generate feature vectors. Recurrent NN (RNN) is a particular kind of Artificial NN (ANN) that generates the utilization of sequential data because of directed links amongst units of individual layers. It can be called recurrent as it is carried out in a similar way to all elements from the order. The RNN is capable of storing memory as its present outcome depends upon the preceding computation. However, the RNN has recognized return only some time steps because of the vanishing gradient issue.

**Fig. 2:**
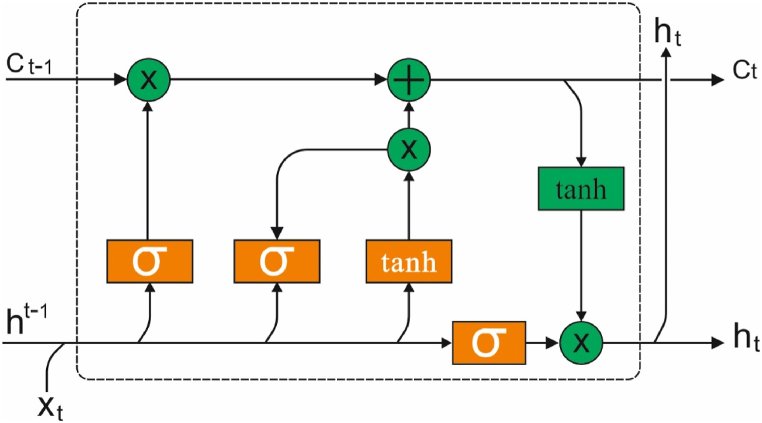
LSTM structure.

As typical RNN undergo vanishing and exploding gradient issues, LSTM was specially planned for overcoming these issues by introducing novel gates that permit an optimum control of the gradient flow and permit optimum maintenance of long‐range dependency. An important module of LSTM has memory cell and gate. Fig. 2 demonstrates the structure of LSTM technique.

These gates from the LSTM cell allow it to preserve a further constant error that backpropagation with time and layer, permitting recurrent net to remain for learning on several time steps [17]. This gate works in tandem for learning and storing long- and short‐term order connected data. The RNN techniques their input order $\left\{x_{1},x_{2},x_{n}\right\}$ utilizing the recurrence:

(1) $$h_{z}=f\left(h_{z-1^{\prime }}x_{z}\right)$$

Where $x_{t}$ refers to the input at z time, and $h_{z}$ signifies the hidden layer (HL). The gates were presented as recurrence functions f to solve the gradient explosion or vanishing issues. The states of LSTM cells are calculated by the following expression:

(2) $$i_{z}=\sigma \left(W_{i}\cdot \left[h_{z-1^{\prime }}x_{z}\right]+b_{i}\right)$$

(3) $$f_{z}=\sigma \left(W_{f}\cdot \left[h_{z-1^{\prime }}x_{z}\right]+b_{f}\right)$$

(4) $$o_{z}=\sigma \left(W_{o}\cdot \left[h_{z-1^{\prime }}x_{z}\right]+b_{o}\right)$$

(5) $$\tilde{C}=\tanh\left(W_{C}\cdot \left[h_{z-1},x_{z}\right]+b_{C}\right)$$

(6) $$C_{z}=f_{z}⊙C_{z-1}+i_{z}⊙{\tilde{C}}_{z}$$

(7) $$h_{z}=o_{z}⊙\tanh\left(C_{z}\right)$$

During the formulas above, $i_{z},f_{z}$ and $o_{z}$ demonstrate the input, forget, and output gates correspondingly. $W^{\prime }s$ and b's parameters define the LSTM units, $C_{z}$ stands for the present cell state, and $\tilde{C}$ has a novel candidate value for the cell state. In 3 sigmoid functions to $i_{z},f_{z}$ and $o_{z}$ gates, which control the resultant amongst [0, 1] as provided in Eqs. (2), (3), (4). The decision on these 3 gates was based on the present input $x_{z}$ and the preceding outcome $h_{z-1}$. Once the gate has 0, Afterward, the signal is blocked by the gate. The forget gate $f_{z}$ shows that the preceding state, $h_{z-1}$, is capable of passing. An input gate $i_{z}$ chooses that novel data in the input for updating or adding to the cell state [18]. The output gate $o_{z}$ resolves that data to resultant dependent upon the cell states. This gate effort is done in tandem with learning and storing long- and short‐term order compared data.

Memory cell C performs as an accumulator of state data. The upgrade of the old cell state $C_{z-1}$ to a novel cell state $C_{z}$ has been carried out utilizing Eq. (6). It can be a procedure that remains for repeating. The weight and bias were identified by the model to minimize the variances amongst the LSTM outcomes and actual trained instances.

In order to construct a very specific ECG detection technique, the BiLSTM was executed that performs as backward and forward LSTM networks to training instance. Both LSTM networks were connected to same resultant layer to relate total contextual data for all sequence points.

By using the Adamax optimizer, the BiLSTM is hyperparameter tuned. It can be an altered version of Adam optimizing in which the distributed variance was projected ∞. Besides, the maximized weights are defined utilizing Eq. (8) [19]:

(8) $$w_{t}^{i}=w_{t-1}^{i}-\frac{\eta }{v_{t}+\epsilon }\cdot {\hat{m}}_{t}$$

Where:

(9) $${\hat{m}}_{t}=\frac{m_{t}}{1-\beta_{1}^{t}}$$

(10) $$v_{t}=\max\left(\beta_{2}∙v_{t-1},\left|G_{t}\right|\right)$$

(11) $$m_{t}=\beta_{1}m_{t-1}+\left(1-\beta_{1}\right)\;G$$

(12) $$G=\nabla_{w}C\left(w_{t}\right)$$

Where η refers to the rate of learning, $\nabla_{w}C\left(w_{t}\right)$ implies the gradient of weight parameter $w_{t}$ x and y equal labels. $w_{t}$ stands for the weights at t he t step , C(.) denotes the cost functions, and $\beta_{i}$ has been utilized for selecting the data required for the old upgrade, whereas $\beta_{i}\in \left[0,1\right]\text{.}$ $m_{t}$ and $v_{t}$ demonstrates the 1st and 2nd moments [20].

Algorithm 1 Pseudocode of AdaMax

**Table:** 

η: Rate of Learning
$\beta_{1}$, $\beta_{2}\in$[0, 1): Exponential decomposed value to moment candidate
C(w): Cost function with w parameter
$w_{0}$: Primary variable vector
$m_{0}\leftarrow 0$
$u_{0}\leftarrow 0$
i←0 (Execute time step)
while w doesn't converge, do
i←i+1
$m_{i}\leftarrow \beta_{1}∙m_{i-1}+\left(1-\beta_{1}\right)∙\frac{\partial C}{\partial w}\left(w_{i}\right)$
$u_{i}\leftarrow \;\max\left(\beta_{2}∙u_{i-1},\left|\frac{\partial C}{\partial w}\left(w_{i}\right)\right|\right)$
$w_{i+1}\leftarrow w_{i}-\left(\eta /\left(1-\beta_{1}^{i}\right)\right)∙m_{i}/u_{i}$
end while
show $w_{i}$ (end parameters)

### 3.3 Design of SSAE-based classification model

The extracted feature vector was passed into the SSAE model to perform the ECG recognition and classification procedure. DL is a novel domain from ML investigation. Its stimulus lies in the structure and simulates the NNs of the brain to systematic learning. It reproduces the process of the human brain for interpreting data. During this case, the deep infrastructure SSAE was implemented to feature decrease and reform [21]. In SSAE procedures, a further abstract maximum level demonstration features by integrating minimum level features for discovering the distributed feature. The SSAE is an unsupervised network, which is a large-scale non-linear method collected by multi-layer neuron cells where the resultants of present layer neurons were fed to connectivity layer neurons. The SSAE or Sparse AE networks are generally composed of 2 parts, the encoded and decoded parts, in which the encoder network decreases maximum dimension as to minimum dimension attributes. During the coding phase, an initial data x is mapped to HL. This procedure is expressed as:

(13) $$z=\sigma_{1}\left(w_{1}x+b_{1}\right)$$

At this point, $\sigma_{1}$ refers to the non-linear functions, $w_{1}$ represents the weight of the encoder network, and $b_{1}$ signifies the bias. Then, novel data was reconstructed by the decoder network:

(14) $$x^{\prime }=\sigma_{2}\left(w_{2}z+b_{2}\right)$$

Where $w_{2}$ signifies the weight of the decoder network and $b_{2}$ refers to the bias. The drive of SAE is for making the resultant closer to it feasible to input by minimized loss functions [22]:

(15) $$\theta =\arg\;\min\;\left[\frac{1}{n}\sum_{i=1}^{n}L\left(x_{i},x_{i}^{\prime }\right)+\beta \sum_{j=1}^{S_{2}}KL\left(\rho \|\hat{\rho }\right)\right]$$

(16) $$\theta =\frac{1}{2N}\sum_{i=1}^{N}\|x_{i}^{\prime }-x_{i}{\|}^{2}+\beta \sum_{j=1}^{S_{2}}KL\left(\rho \|\hat{\rho }\right)$$

Where N defines the amount of HL nodes, ρ refers to the sparse parameters, β demonstrates the weight of the sparse penalty item, and ${\hat{\rho }}_{j}$ stands for the normal activation value of the HL component. The SSAE network has 2 HLs that the decoder network failed to establish, highlighting the feature decreased operation of the networks. Related to the sparse AE, a vital trained process is for learning the parameters θ=(W,b) that permits the method to have minimal input and output deviation. When the optimum parameter θ is attained, the SSAE produces the function $R^{d_{\chi }}\to R^{d_{h\left(2\right)}}$, which transforms new data to minimum dimension space.

### 3.4 Parameter tuning using DFA

The DFA is utilized to determine the optimum parameters of the SSAE model. The DFA was coined by Mirjalili [23]. The metaheuristic technique dependent upon SI was derived from the static and dynamic performance of dragonflies (DFs) naturally. Exploration and exploitation are the two important phases of optimization. Both phases are demonstrated in DFs, also dynamic or static exploring to food/avoid the enemy. Additional performances were added to this performance from DFA: affecting near food and avoiding the enemy.

(17) $$S_{i}=-\sum_{j=1}^{N}X-X_{j}$$

(18) $$A_{i}=\frac{\sum_{j=1}^{N}V_{j}}{N}$$

(19) $$C_{i}=\frac{\sum_{j=1}^{N}X_{j}}{N}-X$$

(20) $$F_{i}=X^{+}-X$$

(21) $$E_{i}=X^{-}+X$$

During the above formulas, $X^{+}$ and $X^{-}$ demonstrates the food position and enemy sources correspondingly. X denotes the instantaneous place of individual, $X_{j}$ stands for the instant location of $j^{th}$ individuals. N refers to the amount of neighboring individuals and $y_{j}$ defines the speed of $j^{th}$ neighboring individuals.

For updating the place of artificial DFs from the search space and simulating its motion, 2 vectors were regarded as step (*X*) and place (*X*). The step vector is regarded as speed refers to the way of DF indication (Eq. (22)). After, the place vector was upgraded in (Eq. (23)):

(22) $$\nabla X_{t+1}=\left(sS_{i}+aA_{i}+cC_{i}+fF_{i}+eE_{i}\right)+w\nabla X_{t}\text{,}$$

(23) $$X_{t+1}=X_{t}+\nabla X_{t+1}\text{,}$$

Where , a, and c values in Eq. (22) define the separation, alignment, and cohesion co-efficient correspondingly, and f, e, w, and t values signify the food factors, enemy factors, inertia co-efficient, and iteration numbers correspondingly [24]. These co-efficient and declared factors allow the execution of exploratory and exploitative performances. In the dynamic swarms, DF is inclined to align its flights. In the static motion, the alignment was very low, but the appropriate for attacking the enemy was extremely high.

The DFA method resolves an FF to achieve increased classifier efficiency. It describes a positive integer to represent the optimal efficacy of the solution candidate. The decline of the classifier error rate was regarded as FF. A better solution is a minimal error rate, and the worst solution obtains a maximum error rate.

(24) $$fitness\left(x_{i}\right)=ClassifierErrorRate\left(x_{i}\right)=\frac{numerb\;of\;misclassified\;samples}{Total\;number\;of\;samples}\times 100$$

## 4 Experimental validation

This study uses the PTB-XL dataset [25] that has a group of 21 837 ECG records of 10s length in 18 885 patients, where 48 % are female and the remaining 52 % of patients are male. Also, the datasets involve five main classes such as hypertrophy (HYP), conduction disturbance (CD), ST/T changes (STTC), normal ECG (NORM), and myocardial infarction (MI). Only one previous study was conducted on PTX-XL datasets that include Inception-based frameworks and ResNet for ECG signal detection. The parameter setting is shown in the following: activation: ReLU, learning rate 0.1, batch size: 7, dropout: 0.5, epochs: 50. For experimental validation, a 10-fold cross-validation model is exploited for splitting the datasets into training and testing parts.

**Fig. 3:**
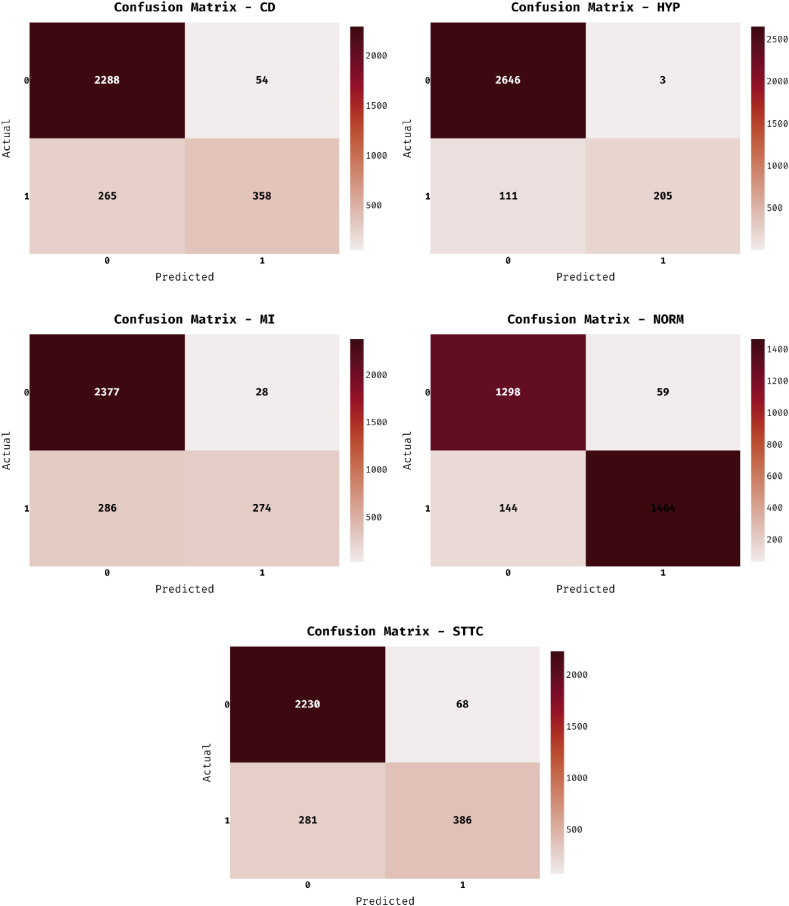
Confusion matrix of ADL-ECGSR method.

The confusion matrices generated by the ADL-ECGSR method on the ECG recognition process are illustrated in Fig. 3 [26]. The figures highlighted that the ADL-ECGSR method has categorized all the instances properly. For example, under the CD class, the ADL-ECGSR method categorized 2288 instances as absent and 358 instances as present classes correspondingly. Eventually, under the MI class, the ADL-ECGSR methodology categorized 2377 instances as absent and 274 instances as present classes correspondingly. Meanwhile, under the NORM class, the ADL-ECGSR methodology categorized 1298 instances as absent and 1464 instances as present classes correspondingly. Finally, under the STTC class, the ADL-ECGSR methodology has ordered 2230 instances to be absent and 386 instances to be present classes correspondingly.

**Fig. 4:**
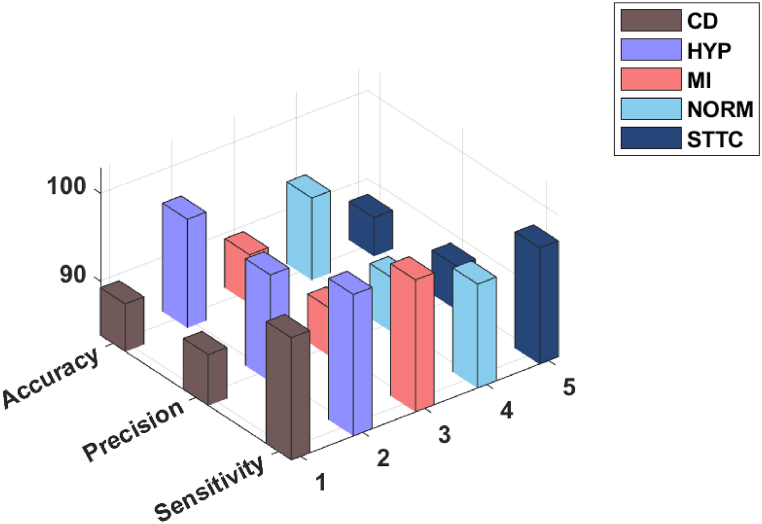
$Accu_{y}$, $prec_{n}$, and $sens_{y}$ analysis of ADL-ECGSR technique.

Table 1 provides a comprehensive analysis of the ADL-ECGSR approach. Fig. 4 illustrates the ECG analysis of the ADL-ECGSR approach interms of $accu_{y}$, $prec_{n}$, $sens_{y}$, ${spec}_{y}$, $F_{score}$, and MCC. The experimental values reported the betterment of the ADL-ECGSR approach interms of different measures. For example, with CD class, the ADL-ECGSR method has attained an $accu_{y}$, $prec_{n}$, $sens_{y}$, ${spec}_{y}$, $F_{score}$, and MCC of 89.24 %, 89.62 %, 97.69 %, 57.46 %, 93.48 %, and 64.96 % correspondingly. Furthermore, with HYP class, the ADL-ECGSR method has attained an $accu_{y}$, $prec_{n}$, $sens_{y}$, ${spec}_{y}$, $F_{score}$, and MCC of 96.16 %, 95.97 %, 99.89 %, 64.87 %, 97.89 %, and 78.24 %. Meanwhile, with MI class, the ADL-ECGSR method has attained an $accu_{y}$, $prec_{n}$, $sens_{y}$, ${spec}_{y}$, $F_{score}$, and MCC of 89.41 %, 89.26 %, 98.84 %, 48.93 %, 93.80 %, and 61.81 % correspondingly. Also, with NORM class, the ADL-ECGSR technique has reached an $accu_{y}$, $prec_{n}$, $sens_{y}$, ${spec}_{y}$, $F_{score}$, and MCC of 93.15 %, 90.01 %, 95.65 %, 91.04 %, 92.75 %, and 86.42 %. Finally, with STTC class, the ADL-ECGSR method has reached an a $ccu_{y}$, $prec_{n}$, $sens_{y}$, ${spec}_{y}$, $F_{score}$, and MCC of 88.23 %, 88.81 %, 97.04 %, 57.87 %, 92.74 %, and 63.67 % correspondingly.

**Table 1:** Result analysis of ADL-ECGSR under various measures.

Measures	$Accu_{y}$	$Prec_{n}$	$Sens_{y}$	$Spec_{y}$	$F_{score}$	MCC
**CD**	89.24	89.62	97.69	57.46	93.48	64.96
**HYP**	96.16	95.97	99.89	64.87	97.89	78.24
**MI**	89.41	89.26	98.84	48.93	93.80	61.81
**NORM**	93.15	90.01	95.65	91.04	92.75	86.42
**STTC**	88.23	88.81	97.04	57.87	92.74	63.67
**Average**	91.24	90.73	97.82	64.03	94.13	71.02

**Fig. 5:**
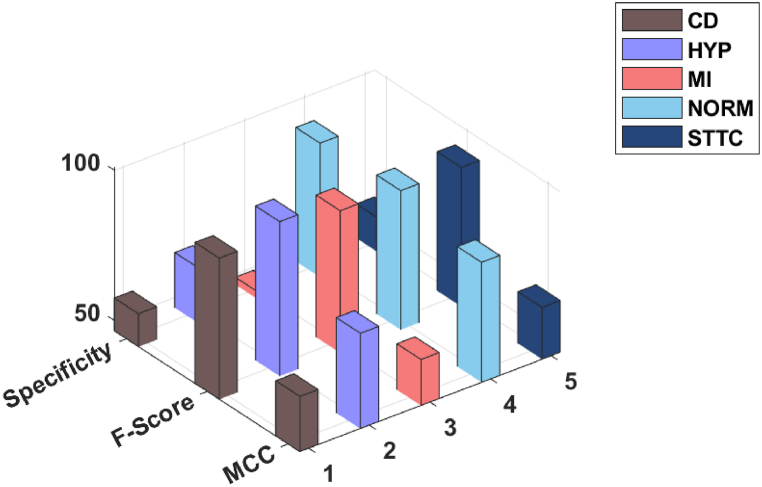
$Spec_{y}$, $F_{score}$, and MCC analysis of ADL-ECGSR technique.

Fig. 5 depicts the ECG analysis of the ADL-ECGSR method with respect to $spec_{y}$, $F_{score}$, and MCC. The experimental values described the betterment of the ADL-ECGSR method in terms of distinct measures. For instance, with CD class, the ADL-ECGSR approach has gained $spec_{y}$, $F_{score}$, and MCC of 57.46 %, 93.48 %, and 64.96 %, respectively. In the meantime, with MI class, the ADL-ECGSR method has reached $spec_{y}$, $F_{score}$, and MCC of 48.93 %, 93.80 %, and 61.81 %, respectively. At last, with STTC class, the ADL-ECGSR methodology has achieved $spec_{y}$, $F_{score}$, and MCC of 57.87 %, 92.74 %, and 63.67 %, correspondingly.

**Fig. 6:**
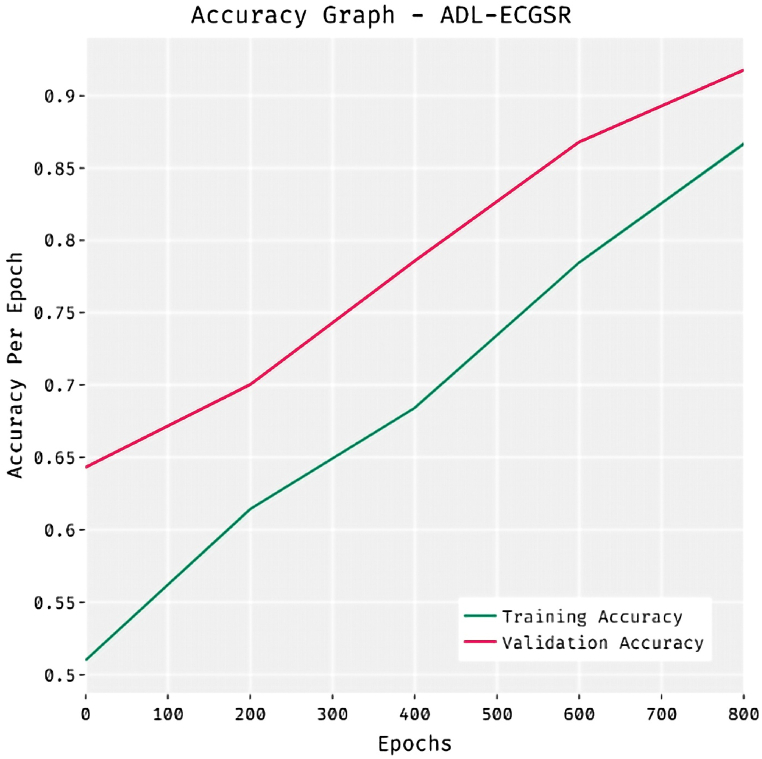
Accuracy graph analysis of ADL-ECGSR method.

Fig. 6 demonstrates the accuracy analysis of the ADL-ECGSR method on the test dataset. The results demonstrated that the ADL-ECGSR technique has obtained maximum performance with the highest training and validation accuracy. The training accuracy steadily improves as the number of epochs rises, stabilizing notably beyond 400 epochs with values constantly above 0.9. This depicts that the network effectively learns and improves its predictions over time, reaching a high training accuracy of 0.89 under 800 epochs. Likewise, the validation accuracy illustrates a steady increase, attaining the highest at 0.86 by 800 epochs, showing the capacity of the model to generalize well on unseen data. The ADL-ECGSR method has obtained maximum validation accuracy over the training accuracy.

**Fig. 7:**
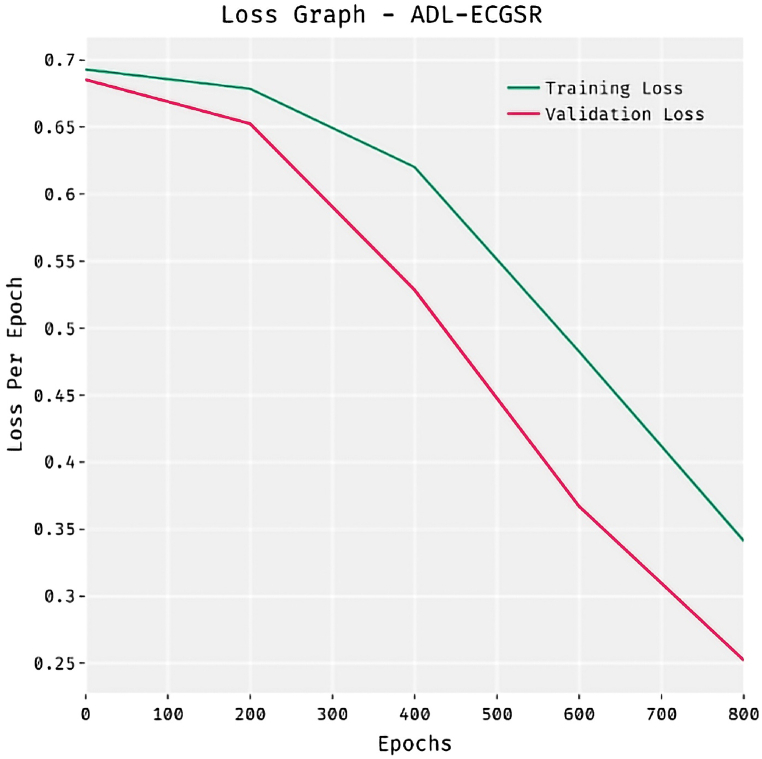
Loss graph analysis of ADL-ECGSR technique.

Fig. 7 illustrates the loss analysis of the ADL-ECGSR method on the test dataset. The outcomes showed that the ADL-ECGSR method has resulted in an effective outcome with decreased training and validation loss. Here, both training and validation curves gradually decrease with epochs. The validation loss stabilizes below 0.5 after 400 epochs, depicting robust learning and generalization abilities. These stable trends propose that the network attains reliable achievement and convergence with extended training, emphasizing the efficiency of the training phase in refining the ADL-ECGSR model. The ADL-ECGSR approach has provided lower validation loss over the training loss.

**Fig. 8:**
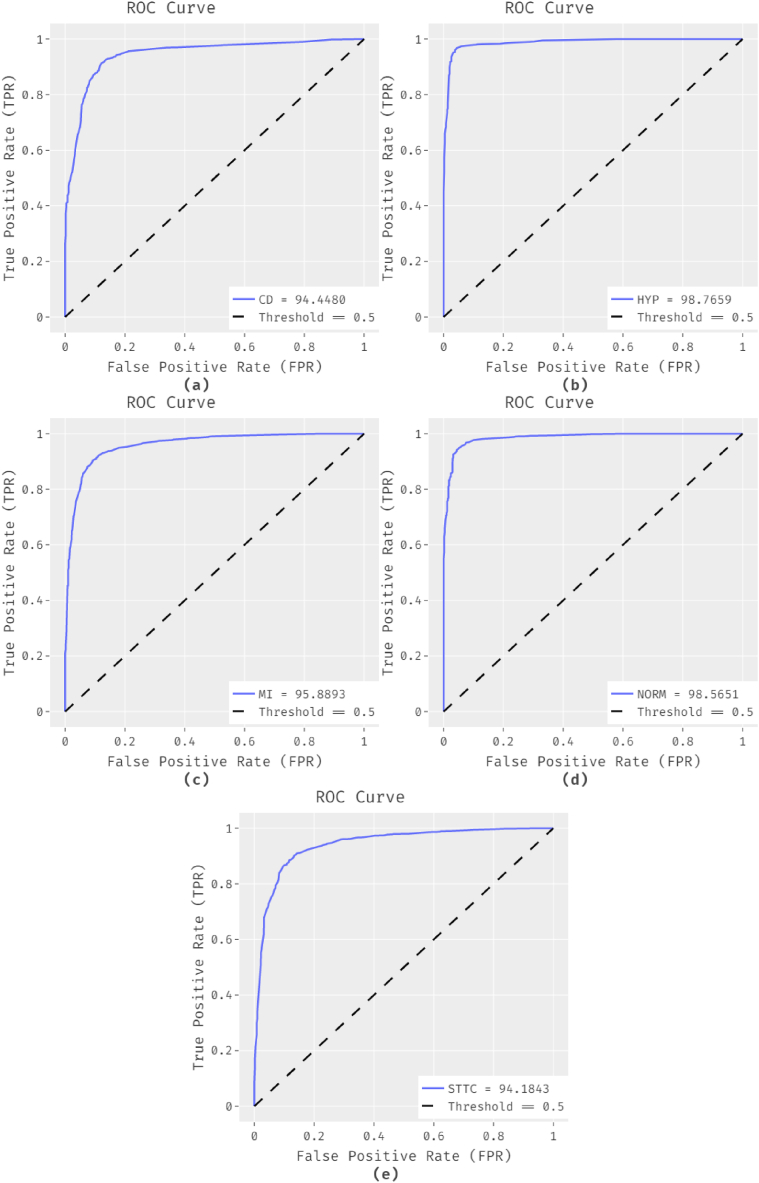
ROC analysis of ADL-ECGSR method.

Fig. 8 depicts the set of ROC analyses of the ADL-ECGSR method on the test dataset. The figures show that the ADL-ECGSR method has resulted in increased values of ROC. For example, the ADL-ECGSR method has categorized the CD class with an ROC of 94.4480. Likewise, the ADL-ECGSR method has ordered the HYP class with an ROC of 98.7659. Next, the ADL-ECGSR methodology has categorized the MI class with an ROC of 95.8893. Along with that, the ADL-ECGSR methodology has categorized the NORM class with an ROC of 98.5651. Lastly, the ADL-ECGSR methodology has ordered the STTC class with an ROC of 94.1843.

**Fig. 9:**
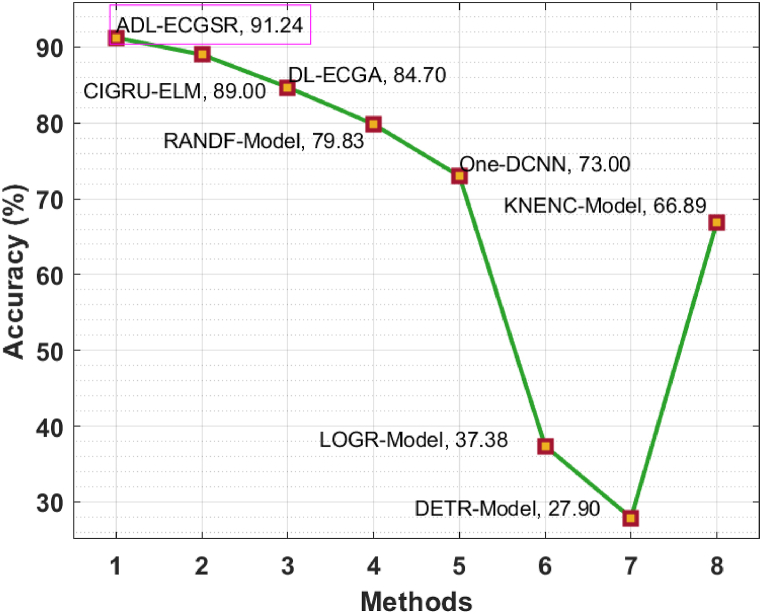
Comparative analysis of ADL-ECGSR method with current methods.

To validate the improvement of the ADL-ECGSR method, a comparative $accu_{y}$ analysis of the ADL-ECGSR method is performed with recent methods CIGRU_ELM, DL_ECGA, Random Forest (RANDF), 1D CNN (One_DCNN), Logistic Regression (LOGR), Decision Tree (DETR), and K-Nearest Neighbour (KNENC) in Table 2 and Fig. 9 [27,28].

**Table 2:** Comparative analysis of ADL-ECGSR under various measures with existing methods.

Methods	Accuracy (%)
ADL-ECGSR	91.24
CIGRU_ELM	89.00
DL_ECGA	84.70
RANDF	79.83
1D_DCNN	73.00
LOGR	37.38
DETR	27.90
KNENC	66.89

The outcomes portrayed that the LOGR and DETR methods have attained reduced $accu_{y}$ values of 37.38 % and 27.90 %, respectively. Next, the 1D-DCNN and KNENC models obtained slightly improved $accu_{y}$ values of 73 % and 66.89 %, respectively. In line with this, the computational intelligence-based GRU (CIGRU) with extreme learning machine (ELM), random forest (RANDF), and DL-ECGA techniques have resulted in maximum $accu_{y}$ values of 89 %, 79.83 %, and 84.70 % correspondingly. However, the ADL-ECGSR methodology has surpassed the other methods with the highest $accu_{y}$ of 91.24 %. From the outcome analysis, it can be ensured that the ADL-ECGSR methodology can achieve improved CVD detection and classification processes.

## 5 Conclusion

This study develops a novel ADL-ECGSR approach for CVD detection and classification. The ADL-ECGSR technique incorporates different stages of operations, namely pre-processing, BiLSTM-based feature extraction, SSAE-based classification, Adamax-based hyperparameter optimization, and DFA-based parameter tuning. The utilization of the Adamax and DFA models has assisted in accomplishing enhanced detection efficiency. The performance analysis of the ADL-ECGSR method takes place against the benchmark PTB-XL dataset, and the outcomes are inspected under various measures. The comparison study shows the remarkable performance of the ADL-ECGSR method over the existing methods with the maximum $accu_{y}$ of 91.24 %. Thus, the ADL-ECGSR method is utilized as a powerful tool for ECG recognition in real-time. In the future, hybrid DL-based ensemble models can be designed to optimize the recognition performance. In addition, improved DFA can be developed by the use of the QOBL concept to enhance the population initialization process. The computational complexity of the proposed ADL-ECGSR technique may limit its real-time applicability in clinical settings. Also, the generalization of the model to diverse patient populations and noise levels should be further investigated to ensure its robustness. The limitations of the ADL-ECGSR technique may comprise the complexity and computational demands related to incorporating several advanced models such as BiLSTM, Adamax optimizer, DFA, and SSAE. Future works may concentrate on addressing these computational threats to improve scalability and effectualness. Furthermore, additional validation on several datasets beyond PTB-XL may strengthen the reliability and generalizability of the technique across diverse populace and healthcare scenarios. Finally, exploring interpretability models to improve comprehension of model decisions in clinical contexts may enhance trust and adoption by medical experts.

## Data availability statement

Data sharing is not applicable to this article as no datasets were generated during the current study.

## Ethics approval

This article does not contain any studies with human participants performed by any of the authors.

## Consent to participate

Not applicable.

## Informed consent

Not applicable.

## CRediT authorship contribution statement

**Hamed Alqahtani:** Writing – review & editing, Writing – original draft, Methodology, Funding acquisition, Data curation, Conceptualization. **Ghadah Aldehim:** Writing – review & editing, Writing – original draft, Validation, Methodology, Investigation. **Nuha Alruwais:** Writing – review & editing, Writing – original draft, Methodology, Data curation, Conceptualization. **Mohammed Assiri:** Writing – review & editing, Writing – original draft, Validation, Resources, Project administration. **Amani A. Alneil:** Writing – review & editing, Writing – original draft, Validation, Methodology. **Abdullah Mohamed:** Writing – review & editing, Visualization, Validation, Software, Methodology.

## Declaration of competing interest

The authors declare that they have no known competing financial interests or personal relationships that could have appeared to influence the work reported in this paper.
